# Elevating hope among children with Attention deficit and hyperactivity disorder through virtual reality

**DOI:** 10.3389/fnhum.2014.00198

**Published:** 2014-05-07

**Authors:** Shimon Shiri, Ariel Tenenbaum, Orly Sapir-Budnero, Isaiah D. Wexler

**Affiliations:** Department of Physical Medicine and Rehabilitation, Hadassah University HospitalJerusalem, Israel

**Keywords:** ADHD, hope, virtual reality, self face recognition, mirror neurons

## Introduction

Children with attention deficit and hyperactivity disorder (ADHD) suffer from a variety of emotional and social difficulties including high levels of depression, anxiety, poor regulation, other executive malfunctions, and lack of satisfying social experiences (Bird et al., 1993; Greene et al., 2001; Gillberg et al., 2004; Barkley, 2006; Klimkeit et al., 2006; Daviss, 2008; Elia et al., 2008; McQuade and Hoza, 2008; Anastopoulos et al., 2011; Larson et al., 2011). Pharmaceutical treatment of ADHD may be effective for ameliorating the manifestations of ADHD but there may also be a need for supplementary psychotherapeutic interventions (Chronis et al., 2006).

Engendering hope is an ancillary supportive therapy that can provide individuals with ADHD the positive coping skills and appropriate psychic framework for handling the challenges that they confront. Hope, in this context, is a psychological construct that relates to the ability of individuals to set themselves meaningful goals, to find channels to reach these goals, and to consistently work toward achieving these goals (Snyder et al., 1991).

A potential tool for instilling hope in patients with ADHD is virtual reality (VR) (Riva, 2005). VR is an advanced form of human-computer interface that allows the user to *interact* with, and feel *present* within a computer generated environment. Similar to Descartes' deceiving god but with nobler intentions, researchers are utilizing models of immersive virtual therapy that enable individuals with physical/mental challenges such as chronic pain to set meaningful goals and visualize alternative realities where their pain or disability is minimized (Magora et al., 2006). For example, Hoffman et al. (2011) have presented VR systems that facilitate distraction thereby reducing perceived pain in individuals undergoing painful medical procedures such as non-pharmacologic analgesic for acute burn pain (Hoffman et al., 2011). In addition, we have recently introduced a combined VR-biofeedback system that allows individuals with chronic headache to learn to reduce their physiological arousal, following which, they can their view virtual image as headache free (Shiri et al., 2012a). Utilizing VR for generating hope in ADHD children whom have significant difficulties in creating and holding images is especially challenging as it based on forming a durable interface with computer-based VR programs.

Recently, we have utilized a self-face recognition paradigm to enhance the verisimilitude of a VR-generated substituted reality (Shiri et al., 2012a, 2013). Self-face recognition programs have been successfully utilized for post-stroke disability and pain reduction. Self-face recognition creates a unique cerebral activity pattern which does not occur even when significant others, such as family members are recognized. Viewing a smile has the potential of activating brain structures that are related to reward and positive emotions. Positive emotions may enhance creative thinking and improve cognitive processes, necessary for effective coping with various challenges (Muehlberger et al., 2011). Although specific relationships between activation of reward structures and positive emotions need to be examined utilizing brain imaging techniques, a recent study suggested that static pictures of emotional facial expressions activate brain structures that are involved in the processing of emotional stimuli (Johnson et al., 2010). Similarly, a sequence of emotional facial expressions changes occur, different brain networks are involved. For example, it was found that the onset of happy and the offset of angry expressions induced significant activation in the left dorsal striatum (Johnson et al., 2010). These findings would suggest that the VR paradigm may be useful for children with ADHD as the interaction of salient brain activity created by self-face recognition together with the activation of reward brain centers produce a platform that is both cerebrally robust and emotionally positive for self-confidence and growth.

## Theoretical background

While the neurocognitive aspects of ADHD have been studied intensely, the secondary emotional, behavioral, and social co-morbid aspects of ADHD are less-well understood. These difficulties include of depression, anxiety, and social adaptation difficulties (Bird et al., 1993; Greene et al., 2001; Gillberg et al., 2004; Barkley, 2006; Klimkeit et al., 2006; Elia et al., 2008; McQuade and Hoza, 2008; Anastopoulos et al., 2011) which can have both short and long-term ramifications. Current interventions for emotional and behavioral symptoms associated with ADHD include pharmacotherapy and psychotherapy. Psychodynamic (Gilmore, 2000, 2002; Conway, 2012) and cognitive behavioral therapy (Safren et al., 2010; Antshel et al., 2011, 2012) are two types of psychotherapeutic approaches that have been utilized for ADHD and shown to be beneficial. The drawback of psychotherapy is that it is expensive, not always available, and may be challenging in the setting of ADHD in which children have difficulty in focusing. There is a need for novel approaches toward treating the secondary, but often devastating manifestations of ADHD. Here we suggest a cost-effective and immediately accessible therapeutic paradigm that utilizes VR to create hope in the form of vivid therapeutic and optimistic self images.

Hope is defined in terms of agency and pathways (Snyder et al., 1991). Agency relates to the motivational components necessary for consistent and sustained efforts that are requisite for achieving goals. Pathways refer to the perceived methods for achieving established goals. Hope has been shown to be a significant factor in coping with various difficult or challenging situations. For example, high levels of hope predict better academic performance (Rand, 2009) and self-efficacy (Davidson et al., 2012). In a recent work (Shiri et al., 2012b), we have found that hope is associated with improved mental and physical health parameters among individuals with post-polio syndrome who are generally in poor physical and mental health due to the residual effects of polio. While little can be done to change the physical limitations associated with the post-polio syndrome, those with higher levels of hope perceived themselves to be healthier and have a greater quality of life.

In light of these findings, we plan to examine the effect of elevating levels of hope among children with ADHD as a method for improving their coping skills as assessed in specific emotional, behavioral, and social domains. Setting meaningful goals, finding ways to reach these goals and achieving the necessary motivation are processes that by themselves are rewarding and increase the chance of attaining set upon goals.

Instilling hope has been traditionally achieved through psychotherapy and specific goal setting as part of cognitive behavioral therapy. A main element of this therapeutic approach is to provide accessible pathways for achieving the goals and providing the appropriate motivation that encourages patients to strive toward accomplishing set upon goals. Similarly, VR is a technique which allows users to visualize goals in a vivid and sharp manner. The verisimilitude inherent in VR allows for viewing specific goals as though they were real. This is particularly important for children with ADHD, given the difficulties these children often experience in imaging, which is a necessary prerequisite for increasing hope (Abraham et al., 2006).

Regarding the ability to visualize goals, VR can compensate for the limited ability of humans to imagine and keep desired images actively. A continuous re-activation of the visual representations is required to keep the image within visual memory (Kosslyn et al., 2006). Visualizing goals is particularly difficult and often impossible for individuals with a wide range of chronic limitations including ADHD (Abraham et al., 2006). VR can compensate for these limitations by providing the desired scenes through the use of specially constructed software programs. The sense of presence (the sense of “being there”) is efficiently achieved by VR, and thereby serves as a potent replacement to guided imagery by providing expressive optimistic images even to individuals with difficulty to follow imagery suggestions (Riva, 2003).

ADHD is diagnosed mainly at a young age, and most of the patients are children and adolescents. Such computerized technologies are particularly appealing to this age group which in turn increases their enthusiasm and adherence to treatment.

## Preliminary studies

In previous work related to stroke rehabilitation (Hoffman et al., 2011) and coping with chronic headache in children (Shiri et al., 2012a), we adopted a self-face recognition VR paradigm. The perspective embodied in this paradigm is different from other VR systems which provide a first person (egocentric) perspective. This paradigm was implemented in light of recent neuropsychological findings suggesting that self-face recognition is faster and more accurate than recognition of strange faces or even highly familiar faces and is characterized by unique bilateral activity (Keyes and Brady, 2010). It has been suggested that self-face recognition creates a unique activation of limbic structures in the right hemisphere in conjunction with left-sided associative and executive regions and that that produces a significant experience of self-awareness (Kircher et al., 2001).

In a pilot study of post-stroke patients, we tested the feasibility and efficacy of a novel motion capture VR system that allows for integrated self-face viewing and mirror visual feedback. This potential rehabilitation tool was tested on 6 post-stroke patients with paretic upper limbs. The system via the novel interface between the patient and the VR system allowed for the replacement of the impaired arm by a virtual arm. Upon making small movements of the paretic arm, patients viewed themselves virtually performing healthy full-range movements. Each patient received 10 sessions of treatment. During the duration of the therapeutic intervention, all participants succeeded in learning how to operate the system. Subject performance within the virtual environment and a set of clinical-functional measures recorded before the VR treatment, at 1 week and after 3 months indicated neurological status and general functioning improvement as shown by a variety of parameters (Hoffman et al., 2011). For example, patients were assessed as to their ability to pick up a fruit, and all 6 improved. Objective measurements of function also showed before and after intervention (Table 1). Although there was no control group and the numbers were small, the findings were sufficiently encouraging for our group to proceed with a second study.

In the subsequent study of pediatric headache (migraine and tension), ten children participated in a single arm prospective study to determine the efficacy of a combined intervention consisting of biofeedback based techniques of relaxation coupled to a VR system. The VR algorithm was designed so patients were led by their virtual self from a painful state to a relaxed pain free state. Biofeedback was based on a reduction of galvanic skin response which is a measure of relaxation. The subjects underwent 10 sessions, and at the end, several outcomes were measured including measurements of pain, quality of life, and a survey related to the efficacy of the intervention (Shiri et al., 2012a). Selected parameters are shown in Table 2. Subjects felt that the intervention was helpful and most would recommend the treatment to others. Quality of life and perceived severity of headache measured before and after the intervention were significantly improved, and perceived limitations on activity related to headache pain was also mitigated (close to statistical significance).

## Conclusion

Based on the preliminary results from these pilot studies, we suggest that implementing a self-face viewing paradigm may be particularly effective in drawing patients' attention and in generating sound neural processing of the virtual scenery, especially neural networks within the mirror neuron system (Uddin et al., 2005). Both the subjective improvement in hand function among stroke patients and the reduction of pain in chronic headache suggest that VR could have a role in instilling hope in individuals with ADHD and facilitate behavioral changes.

The virtual classroom used for evaluation and treatment of ADHD is a familiar and well studied model used to examine various neuropsychological abilities in an environment that simulates a real classroom (Rizzo et al., 2006). In the paradigm that is being developed, we propose that children with ADHD view themselves as achievers of significant, yet realistic goals. Measureable outcomes of this paradigm will be increasing levels of motivation and self-efficacy as assessed in various learning, behavioral, and social settings. Examples of third person VR that can be employed include programs for children who are easily distracted in the classroom setting. These children can view themselves sitting and focusing on specific tasks assigned by the teacher. Children who are disruptive and have explosive behavior at home can view themselves as sitting and doing homework in an orderly and structured manner. The challenge in developing specific VR programs is to identify behaviors that are amenable to modification and are within the capabilities of the children with ADHD.

In future studies we would like to examine the utility of a VR-based intervention to enhance the self-image of children and adolescents with ADHD. Picturing oneself performing a desired behavior from a third-person perspective has the potential of causing individuals to adopt attitudes that are concordant and harmonious with the content drawn in the pictures (Libby et al., 2007). This in turn provides the subjects with hope that they can achieve specific goals and this enhances self-confidence and motivation. Positive emotions produced by viewing a human smile induce a reduction in physiological and psychological stress as shown by Kraft et al. (Kraft and Pressman, 2012), who showed that smiling participants had lower heart rates during stress recovery as compared to controls. Higher levels of positive emotions are associated with greater engagement with the coping process, and individuals are more likely to think through their behavioral options before acting (Tugade et al., 2004).

In conclusion, elevating hope among children with ADHD utilizing a self- face recognition paradigm specifically designed for the needs of children with ADHD has the potential for providing an emotionally positive experience that is therapeutically beneficial in treating the cognitive impairments.

### Conflict of interest statement

The authors declare that the research was conducted in the absence of any commercial or financial relationships that could be construed as a potential conflict of interest.

## References

[B1] Abraham A. Windmann S. Siefen R. Daum I. Güntürkün O. (2006). Creative thinking in adolescents with attention deficit hyperactivity disorder (ADHD). Child Neuropsychol. 12, 111–123 10.1080/09297040500320691 16754532

[B2] Anastopoulos A. D. Smith T. F. Garrett M. E. Morrissey-Kane E. Schatz N. K. Sommer J. L. (2011). Self-regulation of emotion, functional impairment, and comorbidity among children with ADHD. J. Atten. Disord. 15, 583–592 10.1177/1087054710370567 20686097 PMC3355528

[B3] Antshel K. M. Faraone S. V. Gordon M. (2012). Cognitive behavioral treatment outcomes in adolescent ADHD. J. Atten. Disord. [Epub ahead of print]. 10.1177/1087054712443155 22628140

[B4] Antshel K. M. Hargrave T. M. Simonescu M. Kaul P. Hendricks K. Faraone S. V. (2011). Advances in understanding and treating ADHD. BMC Med. 9:72 10.1186/1741-7015-9-72 21658285 PMC3126733

[B5] Barkley R. A. (2006). Attention-Deficit/hyperactivity Disorder: A Handbook for Diagnosis and Treatment. New York, NY: Guilford Press

[B6] Bird H. R. Gould M. S. Staghezza B. M. (1993). Patterns of diagnostic comorbidity in a community sample of children aged 9 through 16 years. J. Am. Acad. Child Adolesc. Psychiatry 32, 361–368 10.1097/00004583-199303000-00018 8444766

[B7] Chronis A. M. Jones H. A. Raggi V. L. (2006). Evidence-based psychosocial treatments for children and adolescents with attention-deficit/hyperactivity disorder. Clin. Psychol. Rev. 26, 486–502 10.1016/j.cpr.2006.01.002 16483703

[B8] Conway F. (2012). Psychodynamic psychotherapy of ADHD: a review of the literature. Psychotherapy 49, 404–417 10.1037/a0027344 22448924

[B9] Davidson O. B. Feldman D. B. Margalit M. A. (2012). Focused intervention for 1st-year college students: promoting hope, sense of coherence, and self-efficacy. J. Psychol. 146, 333–352 10.1080/00223980.2011.634862 22574424

[B10] Daviss W. B. (2008). A review of comorbid depression in pediatric ADHD: etiology, phenomenology, and treatment. J. Child Adolesc. Psychopharmacol. 18, 565–571 10.1089/cap.2008.032 19108661 PMC2699665

[B11] Elia J. Ambrosini P. Berrettini W. (2008). ADHD. characteristics: I. Concurrent comorbidity patterns in children and adolescents. Child Adolesc. Psychiatry Ment. Health 2:15 10.1186/1753-2000-2-15 18598351 PMC2500004

[B12] Gillberg C. Gillberg I. C. Rasmussen P. Kadesjö B. Söderström H. Råstam M. (2004). Coexisting disorders in ADHD: implications for diagnosis and intervention. Eur. Child Adolesc. Psychiatry 13(Suppl. 1), 180–192 10.1007/s00787-004-1008-4 15322959

[B13] Gilmore K. (2002). Diagnosis, dynamics, and development: considerations in the psychoanalytic assessment of children with AD/HD. Psychoanal. Inq. 22, 372–390 10.1080/07351692209348993

[B14] Gilmore K. A. (2000). Psychoanalytic perspective on attention-deficit/hyperactivity disorder. J. Am. Psychoanal. Assoc. 48, 1259–1293 10.1177/00030651000480040901 11212190

[B15] Greene R. W. Biederman J. Faraone S. V. Monuteaux M. C. Mick E. DuPre E. P. (2001). Social impairment in girls with ADHD: patterns, gender comparisons, and correlates. J. Am. Acad. Child Adolesc. Psychiatry. 40, 704–710 10.1097/00004583-200106000-00016 11392349

[B16] Hoffman H. G. Chambers G. T. Meyer W. J. 3rd. Arceneaux L. L. Russell W. J. Seibel E. J. (2011). Virtual reality as an adjunctive non-pharmacologic analgesic for acute burn pain during medical procedures. Ann. Behav. Med. 41, 183–191 10.1007/s12160-010-9248-7 21264690 PMC4465767

[B17] Johnson K. J. Waugh C. E. Fredrickson B. L. (2010). Smile to see the forest: facially expressed positive emotions broaden cognition. Cogn. Emot. 24, 299–321 10.1080/02699930903384667 23275681 PMC3530173

[B18] Keyes H. Brady N. (2010). Self-face recognition is characterized by “bilateral gain” and by faster, more accurate performance which persists when faces are inverted. Q. J. Exp. Psychol. 63, 840–847 10.1080/17470211003611264 20198537

[B19] Kircher T. Senior C. Phillips M. Rabe-Hesketh S. Benson P. Bullmore E. (2001). Recognizing one's own face. Cognition 78, 1–15 10.1016/S0010-0277(00)00104-9 11062324

[B20] Klimkeit E. Graham C. Lee P. Morling M. Russo D. Tonge B. (2006). Children should be seen and heard: self-report of feelings and behaviors in primary-school-age children with ADHD. J. Atten. Disord. 10, 181–191 10.1177/1087054706289926 17085628

[B21] Kosslyn S. M. Thompson W. L. Ganis G. (2006). The Case for Mental Imagery. New York, NY: Oxford University Press 10.1093/acprof:oso/9780195179088.001.0001

[B22] Kraft T. L. Pressman S. D. (2012). Grin and bear it: the Influence of manipulated facial expression on the stress response. Psychol. Sci. 23, 1372–1378 10.1177/0956797612445312 23012270

[B23] Larson K. Russ S. A. Kahn R. S. Halfon N. (2011). Patterns of comorbidity, functioning, and service use for US children with ADHD. Pediatrics 127, 462–470 10.1542/peds.2010-0165 21300675 PMC3065146

[B24] Libby L. K. Shaeffer E. M. Eibach R. P. Slemmer J. A. (2007). Picture yourself at the polls: visual perspective in mental imagery affects self-perception and behavior. Psychol. Sci. 18, 199–203 10.1111/j.1467-9280.2007.01872.x 17444910

[B25] Magora F. Cohen S. Shochina M. Dayan E. (2006). Virtual reality immersion method of distraction to control experimental ischemic pain. Isr. Med. Assoc. J. 8, 261–265 16671363

[B26] McQuade J. D. Hoza B. (2008). Peer problems in Attention Deficit Hyperactivity Disorder: current status and future directions. Dev. Disabil. Res. Rev. 14, 320–324 10.1002/ddrr.35 19072753

[B27] Muehlberger A. Wieser M. J. Gerdes A. B. M. Frey M. C. M. Weyers P. Pauli P. (2011). Stop looking angry and smile, please: start and stop of the very same facial expression differentially activate threat- and reward-related brain networks. Soc. Cogn. Affect. Neurosci. 6, 321–329 10.1093/scan/nsq039 20460301 PMC3110429

[B28] Rand K. L. (2009). Hope and optimism: latent structures and influences on grade expectancy and academic performance. J. Pers. 77, 231–260 10.1111/j.1467-6494.2008.00544.x 19076999

[B29] Riva G. (2003). Virtual environments in clinical psychology. Psychotherapy 40, 68–76 10.1037/0033-3204.40.1-2.68

[B30] Riva G. (2005). Virtual reality in psychotherapy: review. Cyberpsychol. Behav. 8, 220–230 discussion: 231–240. 10.1089/cpb.2005.8.220 15971972

[B31] Rizzo A. A. Bowerly T. Buckwalter J. G. Klimchuk D. Mitura R. Parsons T. D. (2006). A virtual reality scenario for all seasons: the virtual classroom. CNS Spectr. 11, 35–44 16400254 10.1017/s1092852900024196

[B32] Safren S. A. Sprich S. Mimiaga M. J. Surman C. Knouse L. Groves M. (2010). Cognitive behavioral therapy vs. relaxation with educational support for medication-treated adults with ADHD and persistent symptoms: a randomized controlled trial. JAMA 304, 875–880 10.1001/jama.2010.1192 20736471 PMC3641654

[B33] Shiri S. Feintuch U. Berger I. (2013). A combined virtual reality-biofeedback system to cope with chronic headach. Pain Med. 14, 621–627 10.1111/pme.12083 23659372

[B34] Shiri S. Feintuch U. Lorber A. Moreh E. Twito D. Tuchner-Arieli M. (2012a). A novel virtual reality system integrating online self-face and mirror visual feedback for stroke rehabilitation. Top. Stroke Rehabil. 19, 277–286 10.1310/tsr1904-277 22750957

[B35] Shiri S. Wexler I. D. Feintuch U. Meiner Z. Schwartz I. (2012b). Post-polio syndrome: impact of hope on quality of life. Disabil. Rehabil. 34, 824–830 10.3109/09638288.2011.623755 22149715

[B36] Snyder C. R. Harris C. Anderson J. R. Holleran S. A. Irving L. M. Sigmon S. T. (1991). The will and the ways: development and validation of an individual-differences measure of hope. Pers. Soc. Psychol. 60, 570–85 10.1037/0022-3514.60.4.570 2037968

[B37] Tugade M. M. Fredrickson B. L. Barrett L. F. (2004). Psychological resilience and positive emotional granularity: examining the benefits of positive emotions on coping and health. J. Pers. 72, 1161–1190 10.1111/j.1467-6494.2004.00294.x 15509280 PMC1201429

[B38] Uddin L. Q. Kaplan J. T. Molnar-Szakacs I. Zaidel E. Iacoboni M. (2005). Self-face recognition activates a frontoparietal “mirror” network in the right hemisphere: an event-related fMRI study. Neuroimage 25, 926–935 10.1016/j.neuroimage.2004.12.018 15808992

