# Supplementary material for: Elevating hope among children with Attention deficit and hyperactivity disorder through virtual reality
Source: Front Hum Neurosci. 2014 May 7;8:198. doi: 10.3389/fnhum.2014.00198 (PMC4019862; doi:10.3389/fnhum.2014.00198)
Supplement: Supplementary file 1 [file DataSheet1.PDF]

Table 1: Wilcoxon and Friedman tests of 6 different scales used in the study to evaluate the function of the affected arm and pain

**Evaluation**	**Pre treatment**	**Post treatment**	**3 month follow-up**	**P value for Wilcoxon test**
FM	35.5±12.07	43.17±13.55	45.60±15.43	0.027*
WMFT	49.55±41.89	33.96±45.38	33.73±49.85	0.043*
MAL	1.94±0.73	2.83±1.32	1.91±0.37	0.043*
B&B	9.67±10.63	20.17±16.58	19.00±14.21	0.043*
DY	24.67±17.57	35.17±24.13	37.20±24.90	0.026*

Values are mean ± SD. B&B = Box and Block; DY = dynamometer; FM = upper-extremity portion of the Fugl-Meyer Assessment; MAL = Motor Activity Log; WMFT = Wolf Motor Function Test.

*$p < 0.05$
