# Supplementary material for: Elevating hope among children with Attention deficit and hyperactivity disorder through virtual reality
Source: Front Hum Neurosci. 2014 May 7;8:198. doi: 10.3389/fnhum.2014.00198 (PMC4019862; doi:10.3389/fnhum.2014.00198)
Supplement: Supplementary file 2 [file DataSheet2.PDF]

Table 2: Outcome measures for pediatric headache

	Scale*	Pretest	Posttest	P value (Post vs. Pre)
To what extent does your headache limit your daily function?	0-10	5.00±3.08	2.22±2.99	0.069
On average, how severe was your headache during the previous week?	0-10	4.28±2.92	3.11±2.63	0.015
Pediatric quality of life	23-115	48.67±10.83	39.78±10.24	0.006
Do you feel the treatment helped you?	1-5	-	4.1±1.3	-
To what extent would you recommend it to a friend suffering from a similar problem?	1-5	-	4.1±1.6	-

* values plus standard deviation
